# 2-(4-Isobutyl­phen­yl)-1-(morpholin-4-yl)propan-1-one

**DOI:** 10.1107/S1600536812033442

**Published:** 2012-08-04

**Authors:** Nasirullah, Nazar Ul Islam, M. Nawaz Tahir, Ikhtiar Khan, Muhammad Zulfiqar

**Affiliations:** a Institute of Chemical Sciences, University of Peshawar, Peshawar, Pakistan; b University of Sargodha, Department of Physics, Sargodha, Pakistan

## Abstract

In the title compound, C_17_H_25_NO_2_, the morpholine ring adopts a chair conformation. The benzene ring makes a dihedral angle of 39.81 (13)° with the basal plane of the morpholine group.

## Related literature

For related structures, see: Hansen *et al.* (2003 ▶); Nasirullah *et al.* (2012 ▶).
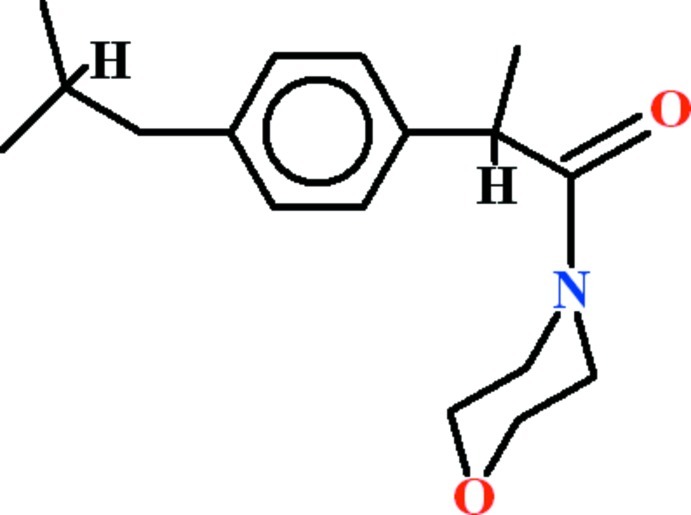


## Experimental

### 

#### Crystal data

C_17_H_25_NO_2_
*M* *_r_* = 275.38
Monoclinic, 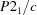

*a* = 14.1389 (19) Å
*b* = 10.3358 (15) Å
*c* = 11.3552 (15) Å
β = 103.426 (8)°
*V* = 1614.1 (4) Å^3^
*Z* = 4
Mo *K*α radiation
μ = 0.07 mm^−1^
*T* = 296 K
0.32 × 0.14 × 0.12 mm

#### Data collection

Bruker Kappa APEXII CCD diffractometer
Absorption correction: multi-scan (*SADABS*; Bruker, 2005 ▶) *T*_min_ = 0.977, *T*_max_ = 0.990
11200 measured reflections
2835 independent reflections
1246 reflections with *I* > 2σ(*I*)
*R*_int_ = 0.100

#### Refinement

*R*[*F* ^2^ > 2σ(*F* ^2^)] = 0.062
*wR*(*F* ^2^) = 0.159
*S* = 0.98
2835 reflections
185 parameters
1 restraint
H-atom parameters constrained
Δρ_max_ = 0.17 e Å^−3^
Δρ_min_ = −0.16 e Å^−3^

### 

Data collection: *APEX2* (Bruker, 2009 ▶); cell refinement: *SAINT* (Bruker, 2009 ▶); data reduction: *SAINT*; program(s) used to solve structure: *SHELXS97* (Sheldrick, 2008 ▶); program(s) used to refine structure: *SHELXL97* (Sheldrick, 2008 ▶); molecular graphics: *ORTEP-3 for Windows* (Farrugia, 1997 ▶) and *PLATON* (Spek, 2009 ▶); software used to prepare material for publication: *WinGX* (Farrugia, 1999 ▶) and *PLATON* (Spek, 2009 ▶).

## Supplementary Material

Crystal structure: contains datablock(s) global, I. DOI: 10.1107/S1600536812033442/wn2484sup1.cif

Structure factors: contains datablock(s) I. DOI: 10.1107/S1600536812033442/wn2484Isup2.hkl

Supplementary material file. DOI: 10.1107/S1600536812033442/wn2484Isup3.cml

Additional supplementary materials:  crystallographic information; 3D view; checkCIF report

## Figures and Tables

**Fig. 1.**
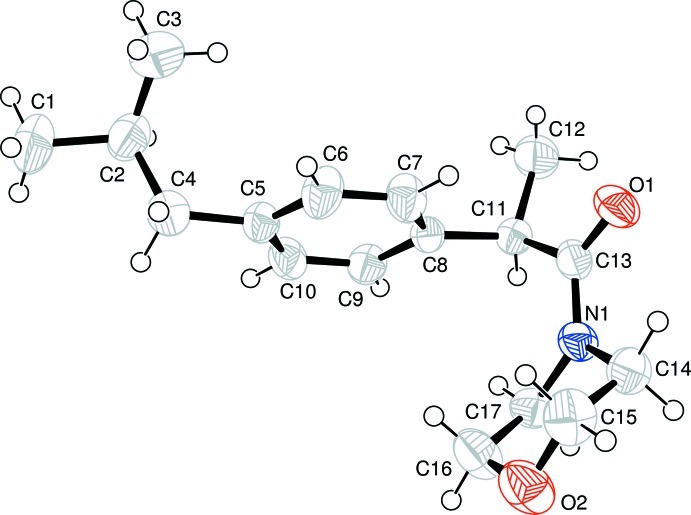
View of the title compound with displacement ellipsoids drawn at the 50% probability level. Hydrogen atoms are shown as spheres of arbitrary radius.

## supplementary crystallographic information

### Comment

The title compound, (Fig. 1) has been synthesized for the purpose of biological studies. The crystal structure of *S*(+)-2-(4-isobutylphenyl)propionic acid (Hansen *et al.*, 2003) has been published; this is related to the present structure by replacement of the carboxyl OH group by the morpholine ring. Also, we have recently submitted to this journal the crystal structure of 2-(6-methoxynaphthalen-2-yl)-1-morpholinopropan-1-one (Nasirullah *et al.*, 2012).

In the title compound, the benzene ring A (C5–C10) and the basal plane B (C14–C17) of the morpholine ring make a dihedral angle of 39.81 (13)°. The morpholine ring adopts a chair conformation, in which atoms N1 and O2 are at distances of -0.623 (5) Å and 0.652 (5) Å, respectively, from the basal plane B.

The crystal structure does not exhibit hydrogen bonding.

### Experimental

A mixture of morpholine (0.54 g, 6.2 mmol) and ibuprofen acid chloride (0.7 g, 3.1 mmol) in 15 ml of dichloromethane was stirred at room temperature for 3 h. After completion of the reaction, the reaction mixture was filtered and the solvent was evaporated, resulting in a crude product which was purified by flash chromatography. Evaporation of solvent yielded the title compound as white crystalline needles. Yield: 84.0%.

### Refinement

The H atoms were positioned geometrically and refined as riding: Csp^2^—H = 0.93 Å, Cmethyl—H = 0.96 Å, Cmethylene—H = 0.97 Å and Cmethine—H = 0.98 Å. *U*_iso_(H) = *xU*_eq_(C), where *x* = 1.5 for methyl H and *x* = 1.2 for all other H atoms.

### Crystal data

**Table:**

C_17_H_25_NO_2_	*F*(000) = 600
*M**_r_* = 275.38	*D*_x_ = 1.133 Mg m^−^^3^
Monoclinic, *P*2_1_/*c*	Mo *K*α radiation, λ = 0.71073 Å
Hall symbol: -P 2ybc	Cell parameters from 1246 reflections
*a* = 14.1389 (19) Å	θ = 1.5–25.0°
*b* = 10.3358 (15) Å	µ = 0.07 mm^−^^1^
*c* = 11.3552 (15) Å	*T* = 296 K
β = 103.426 (8)°	Needle, white
*V* = 1614.1 (4) Å^3^	0.32 × 0.14 × 0.12 mm
*Z* = 4	

### Data collection

**Table:**

Bruker Kappa APEXII CCD diffractometer	2835 independent reflections
Radiation source: fine-focus sealed tube	1246 reflections with *I* > 2σ(*I*)
Graphite monochromator	*R*_int_ = 0.100
Detector resolution: 8.20 pixels mm^-1^	θ_max_ = 25.0°, θ_min_ = 1.5°
ω scans	*h* = −16→16
Absorption correction: multi-scan (*SADABS*; Bruker, 2005)	*k* = −12→12
*T*_min_ = 0.977, *T*_max_ = 0.990	*l* = −13→13
11200 measured reflections	

### Refinement

**Table:**

Refinement on *F*^2^	Secondary atom site location: difference Fourier map
Least-squares matrix: full	Hydrogen site location: inferred from neighbouring sites
*R*[*F*^2^ > 2σ(*F*^2^)] = 0.062	H-atom parameters constrained
*wR*(*F*^2^) = 0.159	*w* = 1/[σ^2^(*F*_o_^2^) + (0.0542*P*)^2^] where *P* = (*F*_o_^2^ + 2*F*_c_^2^)/3
*S* = 0.98	(Δ/σ)_max_ < 0.001
2835 reflections	Δρ_max_ = 0.17 e Å^−^^3^
185 parameters	Δρ_min_ = −0.16 e Å^−^^3^
1 restraint	Extinction correction: *SHELXL97* (Sheldrick, 2008), Fc^*^=kFc[1+0.001xFc^2^λ^3^/sin(2θ)]^-1/4^
Primary atom site location: structure-invariant direct methods	Extinction coefficient: 0.0053 (13)

### Special details

**Table:**

Geometry. Bond distances, angles *etc*. have been calculated using the rounded fractional coordinates. All su's are estimated from the variances of the (full) variance-covariance matrix. The cell e.s.d.'s are taken into account in the estimation of distances, angles and torsion angles
Refinement. Refinement of *F*^2^ against ALL reflections. The weighted *R*-factor *wR* and goodness of fit *S* are based on *F*^2^, conventional *R*-factors *R* are based on *F*, with *F* set to zero for negative *F*^2^. The threshold expression of *F*^2^ > σ(*F*^2^) is used only for calculating *R*-factors(gt) *etc*. and is not relevant to the choice of reflections for refinement. *R*-factors based on *F*^2^ are statistically about twice as large as those based on *F*, and *R*- factors based on ALL data will be even larger.

### Fractional atomic coordinates and isotropic or equivalent isotropic displacement parameters (Å^2^)

**Table:**

	*x*	*y*	*z*	*U*_iso_*/*U*_eq_	
O1	0.35601 (17)	0.2845 (2)	0.5282 (2)	0.0581 (10)	
O2	0.61970 (19)	0.4915 (2)	0.7983 (2)	0.0731 (12)	
N1	0.4656 (2)	0.3220 (2)	0.7033 (2)	0.0433 (10)	
C1	0.0388 (3)	0.9627 (4)	0.8327 (4)	0.097 (2)	
C2	0.0646 (3)	0.8359 (3)	0.7788 (3)	0.0655 (17)	
C3	0.0182 (3)	0.8282 (4)	0.6453 (4)	0.098 (2)	
C4	0.1743 (3)	0.8188 (3)	0.8061 (3)	0.0567 (17)	
C5	0.2088 (2)	0.6854 (3)	0.7822 (3)	0.0437 (12)	
C6	0.2300 (3)	0.6539 (3)	0.6736 (3)	0.0567 (16)	
C7	0.2579 (3)	0.5313 (3)	0.6491 (3)	0.0540 (14)	
C8	0.2674 (2)	0.4342 (3)	0.7350 (3)	0.0374 (12)	
C9	0.2484 (2)	0.4655 (3)	0.8451 (3)	0.0464 (12)	
C10	0.2196 (3)	0.5887 (3)	0.8674 (3)	0.0497 (14)	
C11	0.2928 (2)	0.2963 (3)	0.7066 (3)	0.0406 (12)	
C12	0.2036 (3)	0.2257 (4)	0.6335 (3)	0.0656 (17)	
C13	0.3738 (3)	0.2981 (3)	0.6387 (3)	0.0429 (14)	
C14	0.5436 (3)	0.3446 (3)	0.6411 (3)	0.0536 (14)	
C15	0.5844 (3)	0.4767 (3)	0.6721 (3)	0.0636 (17)	
C16	0.5431 (3)	0.4735 (4)	0.8584 (3)	0.0660 (16)	
C17	0.4977 (3)	0.3419 (3)	0.8333 (3)	0.0467 (12)	
H1A	−0.03054	0.97255	0.81480	0.1454*	
H1B	0.06708	1.03365	0.79843	0.1454*	
H1C	0.06382	0.96156	0.91889	0.1454*	
H2	0.03796	0.76513	0.81872	0.0784*	
H3A	0.03562	0.74781	0.61355	0.1464*	
H3B	0.04078	0.89886	0.60416	0.1464*	
H3C	−0.05117	0.83309	0.63303	0.1464*	
H4A	0.20066	0.88050	0.75783	0.0682*	
H4B	0.20075	0.83986	0.89056	0.0682*	
H6	0.22532	0.71765	0.61471	0.0681*	
H7	0.27059	0.51341	0.57397	0.0647*	
H9	0.25507	0.40278	0.90515	0.0556*	
H10	0.20703	0.60694	0.94248	0.0595*	
H11	0.31628	0.25010	0.78316	0.0487*	
H12A	0.22052	0.13788	0.61992	0.0984*	
H12B	0.18077	0.26834	0.55704	0.0984*	
H12C	0.15319	0.22633	0.67745	0.0984*	
H14A	0.51844	0.33736	0.55428	0.0646*	
H14B	0.59422	0.28019	0.66586	0.0646*	
H15A	0.63699	0.49156	0.63191	0.0764*	
H15B	0.53433	0.54068	0.64276	0.0764*	
H16A	0.49383	0.53924	0.83179	0.0792*	
H16B	0.56830	0.48364	0.94496	0.0792*	
H17A	0.54465	0.27592	0.86816	0.0561*	
H17B	0.44261	0.33479	0.87038	0.0561*	

### Atomic displacement parameters (Å^2^)

**Table:**

	*U*^11^	*U*^22^	*U*^33^	*U*^12^	*U*^13^	*U*^23^
O1	0.0606 (18)	0.0754 (18)	0.0365 (15)	−0.0023 (14)	0.0078 (12)	−0.0122 (12)
O2	0.069 (2)	0.079 (2)	0.076 (2)	−0.0311 (16)	0.0263 (16)	−0.0203 (15)
N1	0.0424 (19)	0.0540 (19)	0.0348 (16)	−0.0021 (15)	0.0119 (15)	−0.0046 (13)
C1	0.082 (4)	0.076 (3)	0.130 (4)	0.031 (3)	0.018 (3)	−0.027 (3)
C2	0.053 (3)	0.057 (3)	0.084 (3)	0.011 (2)	0.011 (2)	−0.005 (2)
C3	0.077 (4)	0.111 (4)	0.090 (3)	0.027 (3)	−0.011 (3)	−0.020 (3)
C4	0.056 (3)	0.054 (3)	0.059 (3)	0.001 (2)	0.011 (2)	−0.0066 (18)
C5	0.041 (2)	0.046 (2)	0.046 (2)	0.0025 (18)	0.0137 (18)	−0.0063 (19)
C6	0.072 (3)	0.044 (2)	0.058 (3)	0.015 (2)	0.023 (2)	0.0139 (18)
C7	0.074 (3)	0.054 (2)	0.038 (2)	0.011 (2)	0.0209 (19)	0.0030 (19)
C8	0.036 (2)	0.042 (2)	0.035 (2)	0.0020 (16)	0.0101 (15)	−0.0018 (16)
C9	0.052 (2)	0.048 (2)	0.040 (2)	0.0067 (19)	0.0123 (18)	0.0058 (17)
C10	0.059 (3)	0.055 (2)	0.037 (2)	0.003 (2)	0.0149 (18)	−0.0054 (19)
C11	0.039 (2)	0.041 (2)	0.042 (2)	0.0008 (17)	0.0099 (17)	−0.0021 (16)
C12	0.049 (3)	0.068 (3)	0.078 (3)	−0.010 (2)	0.011 (2)	−0.018 (2)
C13	0.049 (3)	0.040 (2)	0.040 (2)	0.0022 (18)	0.0108 (19)	−0.0050 (16)
C14	0.047 (2)	0.068 (3)	0.051 (2)	−0.003 (2)	0.022 (2)	−0.0057 (19)
C15	0.067 (3)	0.065 (3)	0.066 (3)	−0.007 (2)	0.030 (2)	0.006 (2)
C16	0.071 (3)	0.074 (3)	0.057 (2)	−0.020 (2)	0.023 (2)	−0.019 (2)
C17	0.048 (2)	0.054 (2)	0.038 (2)	−0.0046 (18)	0.0096 (17)	0.0034 (16)

### Geometric parameters (Å, º)

**Table:**

O1—C13	1.229 (4)	C1—H1C	0.9600
O2—C15	1.412 (4)	C2—H2	0.9800
O2—C16	1.420 (5)	C3—H3A	0.9600
N1—C13	1.357 (5)	C3—H3B	0.9600
N1—C14	1.460 (5)	C3—H3C	0.9600
N1—C17	1.455 (4)	C4—H4A	0.9700
C1—C2	1.526 (5)	C4—H4B	0.9700
C2—C3	1.508 (6)	C6—H6	0.9300
C2—C4	1.520 (6)	C7—H7	0.9300
C4—C5	1.508 (5)	C9—H9	0.9300
C5—C6	1.374 (5)	C10—H10	0.9300
C5—C10	1.375 (5)	C11—H11	0.9800
C6—C7	1.375 (5)	C12—H12A	0.9600
C7—C8	1.384 (5)	C12—H12B	0.9600
C8—C9	1.377 (5)	C12—H12C	0.9600
C8—C11	1.523 (4)	C14—H14A	0.9700
C9—C10	1.378 (5)	C14—H14B	0.9700
C11—C12	1.525 (5)	C15—H15A	0.9700
C11—C13	1.522 (5)	C15—H15B	0.9700
C14—C15	1.492 (5)	C16—H16A	0.9700
C16—C17	1.503 (5)	C16—H16B	0.9700
C1—H1A	0.9600	C17—H17A	0.9700
C1—H1B	0.9600	C17—H17B	0.9700
			
C15—O2—C16	110.1 (3)	C2—C4—H4A	108.00
C13—N1—C14	120.2 (3)	C2—C4—H4B	108.00
C13—N1—C17	127.5 (3)	C5—C4—H4A	108.00
C14—N1—C17	112.1 (3)	C5—C4—H4B	108.00
C1—C2—C3	111.1 (3)	H4A—C4—H4B	108.00
C1—C2—C4	110.2 (3)	C5—C6—H6	119.00
C3—C2—C4	112.6 (3)	C7—C6—H6	119.00
C2—C4—C5	115.3 (3)	C6—C7—H7	120.00
C4—C5—C6	121.7 (3)	C8—C7—H7	120.00
C4—C5—C10	121.7 (3)	C8—C9—H9	120.00
C6—C5—C10	116.6 (3)	C10—C9—H9	120.00
C5—C6—C7	122.2 (3)	C5—C10—H10	119.00
C6—C7—C8	120.7 (3)	C9—C10—H10	119.00
C7—C8—C9	117.5 (3)	C8—C11—H11	108.00
C7—C8—C11	121.2 (3)	C12—C11—H11	108.00
C9—C8—C11	121.2 (3)	C13—C11—H11	108.00
C8—C9—C10	120.8 (3)	C11—C12—H12A	110.00
C5—C10—C9	122.1 (3)	C11—C12—H12B	110.00
C8—C11—C12	110.9 (3)	C11—C12—H12C	109.00
C8—C11—C13	109.8 (3)	H12A—C12—H12B	109.00
C12—C11—C13	110.8 (3)	H12A—C12—H12C	109.00
O1—C13—N1	121.1 (4)	H12B—C12—H12C	109.00
O1—C13—C11	121.1 (3)	N1—C14—H14A	110.00
N1—C13—C11	117.8 (3)	N1—C14—H14B	110.00
N1—C14—C15	109.1 (3)	C15—C14—H14A	110.00
O2—C15—C14	111.4 (3)	C15—C14—H14B	110.00
O2—C16—C17	111.6 (3)	H14A—C14—H14B	108.00
N1—C17—C16	109.8 (3)	O2—C15—H15A	109.00
C2—C1—H1A	110.00	O2—C15—H15B	109.00
C2—C1—H1B	109.00	C14—C15—H15A	109.00
C2—C1—H1C	109.00	C14—C15—H15B	109.00
H1A—C1—H1B	110.00	H15A—C15—H15B	108.00
H1A—C1—H1C	109.00	O2—C16—H16A	109.00
H1B—C1—H1C	109.00	O2—C16—H16B	109.00
C1—C2—H2	108.00	C17—C16—H16A	109.00
C3—C2—H2	108.00	C17—C16—H16B	109.00
C4—C2—H2	108.00	H16A—C16—H16B	108.00
C2—C3—H3A	109.00	N1—C17—H17A	110.00
C2—C3—H3B	109.00	N1—C17—H17B	110.00
C2—C3—H3C	110.00	C16—C17—H17A	110.00
H3A—C3—H3B	109.00	C16—C17—H17B	110.00
H3A—C3—H3C	109.00	H17A—C17—H17B	108.00
H3B—C3—H3C	109.00		
			
C16—O2—C15—C14	−60.7 (4)	C6—C5—C10—C9	−1.2 (5)
C15—O2—C16—C17	58.9 (4)	C5—C6—C7—C8	−1.1 (6)
C14—N1—C13—O1	6.0 (4)	C6—C7—C8—C9	−0.3 (5)
C14—N1—C13—C11	−170.6 (3)	C6—C7—C8—C11	176.6 (3)
C17—N1—C13—O1	179.1 (3)	C7—C8—C9—C10	1.0 (5)
C17—N1—C13—C11	2.5 (4)	C11—C8—C9—C10	−176.0 (3)
C13—N1—C14—C15	119.2 (3)	C7—C8—C11—C12	−78.8 (4)
C17—N1—C14—C15	−54.9 (3)	C7—C8—C11—C13	43.9 (4)
C13—N1—C17—C16	−120.0 (4)	C9—C8—C11—C12	98.0 (4)
C14—N1—C17—C16	53.6 (4)	C9—C8—C11—C13	−139.3 (3)
C1—C2—C4—C5	167.1 (3)	C8—C9—C10—C5	−0.2 (6)
C3—C2—C4—C5	−68.2 (4)	C8—C11—C13—O1	−100.5 (3)
C2—C4—C5—C6	93.4 (4)	C8—C11—C13—N1	76.1 (3)
C2—C4—C5—C10	−85.9 (4)	C12—C11—C13—O1	22.4 (4)
C4—C5—C6—C7	−177.4 (4)	C12—C11—C13—N1	−161.0 (3)
C10—C5—C6—C7	1.9 (6)	N1—C14—C15—O2	58.2 (4)
C4—C5—C10—C9	178.1 (3)	O2—C16—C17—N1	−55.1 (4)
